# Intrathoracic extramedullary hematopoiesis mimicking intrathoracic tumors: A case report

**DOI:** 10.3892/ol.2014.2051

**Published:** 2014-04-09

**Authors:** BO ZHOU, SHENG YAN, SHUSEN ZHENG

**Affiliations:** Division of Hepatobiliary and Pancreatic Surgery, Department of Surgery, First Affiliated Hospital, School of Medicine, Zhejiang University, Hangzhou, Zhejiang 310003, P.R. China

**Keywords:** extramedullary hematopoiesis, hemolytic anemia, hereditary spherocytosis, tumor-like masses

## Abstract

Extramedullary hematopoiesis (EMH) is a rare disease that is characterized by the presence of hemopoietic tissue outside the bone marrow. The masses that form are usually microscopic and asymptomatic, but occasionally lead to tumor-like masses. A 56-year-old male who initially presented to the First Affiliated Hospital, School of Medicine, Zhejiang University (Hangzhou, China) with upper abdominal pain and jaundice was found to have paravertebral masses in the thorax. Histopathological examination of a computed tomography-guided needle aspiration biopsy of the masses revealed EMH. The current study presents this unusual case, in which EMH was diagnosed by chance in a patient with hereditary spherocytosis. As the intrathoracic EMH was asymptomatic, the patient was discharged with the proviso of regular follow-up examinations. The patient exhibited improved blood cell counts following a splenectomy to reduce the hemolysis and stabilize the thoracic masses. The thoracic masses have been closely followed for one and a half years. A correct diagnosis can thus aid in avoiding unnecessary surgical intervention, particularly in an asymptomatic patient.

## Introduction

Extramedullary hematopoiesis (EMH), occurring as a compensatory mechanism for bone marrow dysfunction, is almost always associated with hemoglobinopathies, including thalassemias, sickle cell disease and hereditary spherocytosis (HS), and myelofibrosis, as well as other bone marrow disorders (1). The most common sites of involvement are the spleen, liver and lymph nodes, however, practically any anatomical site may be involved (2–4). Intrathoracic EMH (TEMH) is a rare condition that is often located in the posteroinferior mediastinum and is usually asymptomatic. Clinically, it is important to distinguish masses caused by EMH from other lesions involving the posterior mediastinum.

The current study presents the case of a patient with HS whose primary complaints were abdominal pain and jaundice. The chest radiograph and thoracic computed tomography (CT) scan incidentally revealed posterior mediastinum paravertebral masses, which were diagnosed as EMH by the CT-guided needle aspiration biopsy. The patient provided written informed consent.

## Case report

A 56-year-old male, with no history of serious diseases, was admitted to the First Affiliated Hospital, School of Medicine, Zhejiang University (Hangzhou, China) due to upper abdominal pain during the previous 10 days. The patient was a long-term drinker and had a family history of HS. A physical examination revealed mild pale conjunctivae, icteric sclera, mild right upper abdominal tenderness without rebound tenderness, a negative Murphy’s sign and marked splenomegaly. The whole blood count showed a hemoglobin level of 91 g/l, a red blood cell count of 2.77×10^12^/l, a mean corpuscular volume of 98.6 fl, a mean corpuscular hemoglobin level of 32.9 pg, a total leukocyte count of 4.3×10^9^/l and a platelet count of 169×10^9^/l. Liver function tests revealed marginally elevated transaminases, with an aspartate aminotransferase level of 44 U/l and an alanine transaminase level of 55 U/l, and increased bilirubin levels, with a total bilirubin level of 85 μmol/l and a direct bilirubin level of 50 μmol/l. The hepatitis serology of the hepatitis B virus surface antigen and tumor markers were negative. The abdominal ultrasonography and CT scan showed common bile duct stones, gall stones, cirrhosis and splenomegaly.

**Figure 1:**
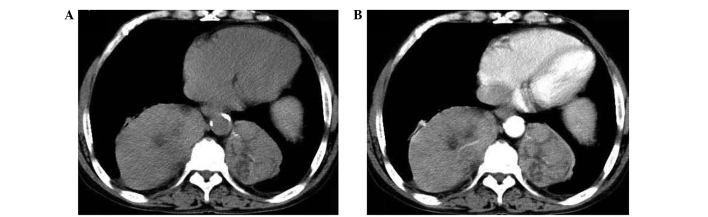
Unenhanced thoracic computed tomography (CT) scan showing (A) multiple well-marginated bilateral solid masses, without calcification, located at the paravertebral region, and (B) lesions showing uneven enhancement during the arterial phase. The necks of the ribs were extremely thin.

**Figure 2:**
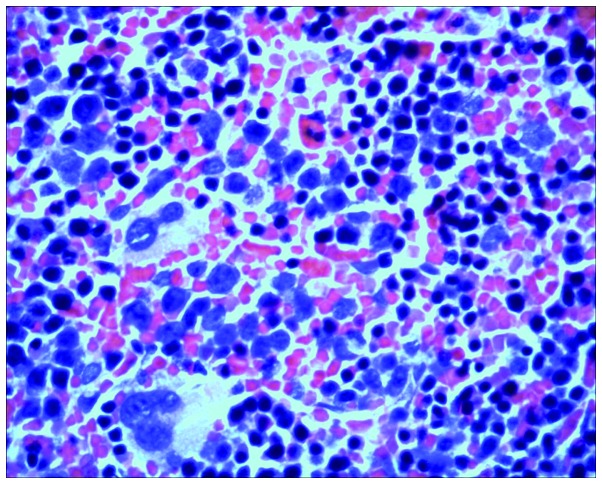
Microscopic examination of a biopsy showing extensive extramedullary hemopoiesis (EMH) with strong erythroid hyperplasia (stain, hematoxylin and eosin; magnification, ×400).

A routine chest radiograph showed bilateral masses of the mediastinum, and a further thoracic CT scan confirmed multiple bilateral smoothly-marginated paravertebral massive tumor-like masses on the posterior mediastinum, with mild enhancement and without bony erosion of the vertebrae or ribs. The largest diameter of the masses was ~11 cm (Fig. 1). A peripheral blood smear showed numerous spherocytes and polychromasia, and the reticulocyte count accounted for 13.3% of the total erythrocytes. A bone marrow aspiration and trephine biopsy analysis demonstrated erythroid hyperplasia only. The osmotic fragility of the red blood cells was also increased. According to these results, the diagnosis of HS was confirmed. For further evaluation, a CT-guided needle aspiration biopsy of the thoracic masses was performed. The histopathological examination presented a diagnosis of EMH, revealing hematopoietic cells with a diffuse distribution, including erythroid, myeloid and megakaryocytic cells (Fig. 2). Next, cholecystectomy was performed using a choledoscope via choledocotomy for exploration. In addition, stone removal, T-tube drainage and splenectomy were performed. As the TEMH was asymptomatic, the patient was discharged with the proviso of regular follow-up examinations being performed.

The patient exhibited improved blood cell counts following a splenectomy to reduce the hemolysis. The thoracic masses have been closely followed for one and a half years and remain stable.

## Discussion

EMH occurs rarely and is characterized by hematopoietic elements that occur outside of the bone marrow. The disease typically develops as a compensatory response in various anemias. The liver, spleen and lymph nodes are the usual sites of EMH, but it may also occur in other sites, including the thymus, kidneys, retroperitoneum, lungs, breasts, skin, brain, adrenal glands and paravertebral areas of the thorax (1–5).

TEMH, particularly posterior mediastinal EMH, is a rare condition that was first described during an autopsy in 1912 (6). The majority of TEMH masses are usually asymptomatic and can be found by microscopic examination, however, occasionally they lead to tumor-like masses, as presented in the current case. Furthermore, TEMH may cause serious complications, including a massive hemothorax, symptomatic pleural effusion, chylothorax or spinal cord compression (7–9). As the manifestation is variable, it is difficult to distinguish EMH from other mediastinal tumors, mainly when the underlying hematologic disease is as yet undiagnosed. For posterior mediastinal masses, such as those identified in the present case, neurogenic tumors, lymphomas, paravertebral abscesses, extrapleural cysts, primary and metastatic malignant neoplasms and mediastinal lymph node hyperplasia must be considered in the differential diagnosis of TEMH (10).

A number of non-invasive diagnostic procedures have been recommended to reach a diagnosis of EMH. These include chest roentgenograms, contrast enhanced CT, magnetic resonance imaging and technetium-99m sulfur colloid radionuclide bone marrow scanning. A study by Chute and Fowler (7) reported that the typical radiographic appearance of TEMH shows unilateral or bilateral, posterior or lateral, well-circumscribed lobulated paravertebral mass lesions, which are usually located caudal to the sixth thoracic vertebrae, without bony erosion and with an absence of calcification. On magnetic resonance imaging, the appearance of EMH is likely to be heterogeneous or homogeneous, depending on the presence of adipose tissue, which is particularly useful in diagnosing intraspinal EMH (9). A study by Hennessy and Salanitri (11) indicated that ^99m^Tc-sulfur colloid or human serum albumin millisphere bone marrow scintigraphy may show increased multiple sites of ectopic paravertebral tracer uptake in the thorax and abdomen, corresponding to the sites of EMH. Tissue biopsy or surgical resection may only be required when the diagnosis of EMH is suspected or when complications require surgical intervention; however, these procedures carry a risk of hemorrhagic complications.

The pathogenesis of TEMH remains unclear, although one explanation is that TEMH may be derived from the extrusion of bone marrow stem cells through the cortex into a subperiosteal location. An additional hypothesis is that TEMH may be derived from embryonic rests or totipotential cells under the persistent stimulus of anemia. In the present case, a few of the necks of the ribs were extremely thin, which may have been responsible for the development of TEMH.

It is usually unnecessary to treat patients with TEMH, with the exception of symptomatic patients. Since extramedullary hematopoietic tissue is highly radiosensitive at relatively small doses, radiotherapy has been indicated to be an effective method for controlling symptomatic spinal cord compression and hemothorax, while surgical treatment is reserved for immediate symptomatic relief. It has also been reported that the surgical resection of EMH may cause further deterioration of anemia and promote hematopoietic behavior in other areas. Therefore, it is important to determine a correct pre-operative diagnosis to avoid unnecessary surgical trauma and improve prognosis.

In conclusion, TEMH must be considered in the differential diagnosis of patients who have chronic anemia with asymptomatic intrathoracic masses. Based on characteristic radiographic observations and chronic hematological conditions, a diagnosis of EMH should be strongly considered and a biopsy may not be necessary.
